# Two Cases of Painful Thyroiditis With Subsequent Hypothyroidism Following Cord Blood Transplant

**DOI:** 10.7759/cureus.57952

**Published:** 2024-04-10

**Authors:** Rika Soga, Yosuke Okada, Kenichi Tanaka, Kenji Koikawa, Yoshiya Tanaka

**Affiliations:** 1 First Department of Internal Medicine, School of Medicine, University of Occupational and Environmental Health, Kitakyushu, JPN

**Keywords:** thyroid goiter, hypothyroidism, thyrotoxicosis, cord blood transplantation, thyroiditis

## Abstract

We report two rare cases of painful thyroiditis approximately 100 days after unrelated cord blood transplantation (CBT), which progressed to hypothyroidism. Patient one, a 45-year-old woman, developed goiter, tenderness, and thyrotoxicity on day 100 after CBT for relapsed acute lymphocytic leukemia. Scintigraphy suggested destructive thyroiditis; symptoms improved with one-month beta-blocker and prednisolone treatment. Two months later, hypothyroidism developed which required supplementation-based treatment. Patient two, a 49-year-old man, developed goiter, tenderness, and thyrotoxicosis on day 96 after CBT for acute myelogenous leukemia. Hypothyroidism developed after nonsteroidal anti-inflammatory drug treatment. Thyroiditis and hypothyroidism should be considered in patients who develop neck pain after CBT.

## Introduction

Allogeneic hematopoietic stem cell transplantation (HSCT) is a curative therapy for serious hematologic conditions, such as leukemia, lymphoma, and aplastic anemia. Bone marrow transplantation has been traditionally the primary source of allogeneic HSCT. However, since the first case of cord blood transplantation (CBT) was performed in Japan in 1997, the number of CBT-HSCT cases has increased considerably. Japan has become the leading country in performing CBT procedures [1]. CBT has several advantages over bone marrow or peripheral blood stem cell graft sources, including less stringent HLA-matching criteria, a lower incidence of chronic graft-versus-host disease (GVHD), and a better steroid response to acute GVHD. This is because cord blood contains a relatively small amount of T cells and a high proportion of naïve T cells [1].

Abnormal thyroid function is a common complication after allogeneic HSCT. Most cases develop hypothyroidism or subclinical hypothyroidism as a late complication [2-4], and total body irradiation (TBI) in the conditioning regimen is thought to be responsible [2]. Although thyroid dysfunction following bone marrow transplantation has been reported previously [2-5], there are only a few reports regarding thyroid abnormalities after CBT. Here, we report two rare cases of painful thyroiditis that occurred approximately 100 days after CBT. The clinical course in both cases extended more than one year and included hypothyroidism.

## Case presentation

Patient one

This patient was a 45-year-old woman who had been diagnosed with acute lymphocytic leukemia at age 37. She received induction therapy according to the Japan Adult Leukemia Study Group (JALSG) treatment protocol ALL-87, as well as JALSG ALL-93-based consolidation, maintenance, and intensification therapy. Hematological remission was achieved and maintained; however, the patient relapsed at age 44. She underwent a nonmyeloablative conditioning regimen with fludarabine, received melphalan 140 mg/m^2^, and underwent 2-Gy of TBI. CBT was performed in April 20XX. Her thyroid function was tested twice before CBT, and the results were within the normal range on both occasions. Cord blood was obtained from an unrelated donor with a human leukocyte antigen (HLA) 6/8 match. GVHD prophylaxis consisted of tacrolimus and 7 mg methotrexate. On day +77, she presented with persistent nausea and was diagnosed with gastrointestinal GVHD grade one. Treatment with prednisolone (PSL; 50 mg/day) and beclomethasone (4 mg/day) was initiated and then tapered gradually. On day +94, PSL was decreased from 5 mg to 2.5 mg, and the patient started to experience fatigue, shortness of breath, palpitation, loss of appetite, and weight loss. On day +100, she developed goiter, neck tenderness, and toughness, particularly over the left thyroid lobe.

Physical examination showed a weight loss of 5 kg in nine days, tachycardia (119 beats/min), hand tremors, and sweating. The C-reactive protein (CRP) level was elevated to 6.33 mg/dL, free triiodothyronine (FT3) was >32.5 pg/mL (normal range, 2.47-4.34), free thyroxine (FT4) was 7.54 ng/dL (normal range, 0.97-1.79), and thyroid-stimulating hormone (TSH) was <0.01 μIU/mL (normal range, 0.61-4.23). These findings were consistent with the diagnosis of thyrotoxicosis. Serological tests were positive for anti-thyroglobulin (Tg) antibody (66 U/mL, normal range, <28 U/mL) but negative for both anti-thyroid peroxidase (TPO) antibody and anti-TSH receptor antibodies (TRAbs). Moreover, adrenocorticotropic hormone and cortisol levels were decreased to <1.5 pg/mL and 3.6 μg/dL, respectively. The symptoms of fatigue and anorexia suggested adrenal insufficiency. Thyroid ultrasound showed diffuse enlargement of the thyroid gland with coarse and heterogeneous parenchyma but no hypoechoic areas often seen in subacute thyroiditis. Parenchymal blood flow was not increased, and no masses were identified. The technetium (^99m^TcO_4-_) scintigraphy uptake rate was 0.69% (normal range, 0.5%-4%), suggesting destructive thyroiditis.

**Figure 1:**
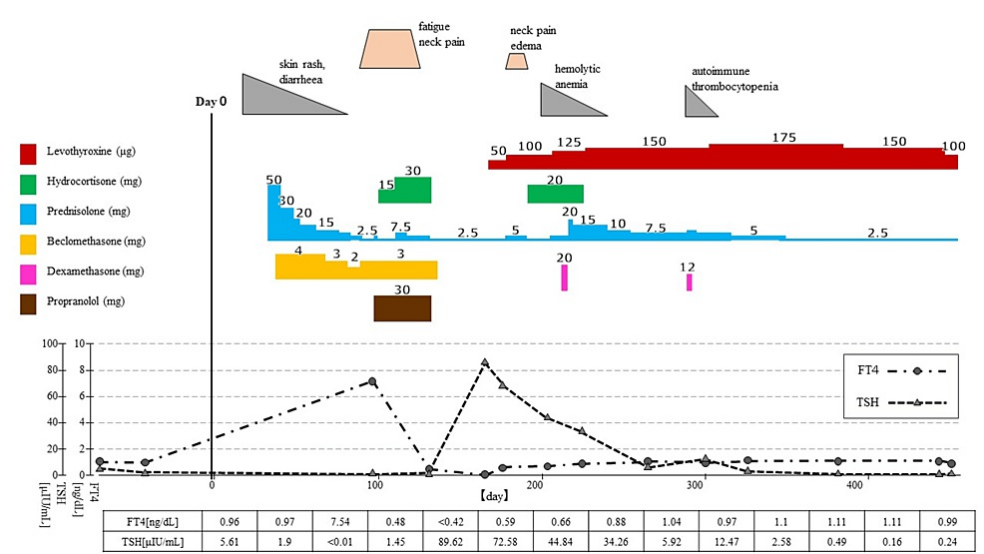
Clinical course of Patient one.

**Figure 2:**
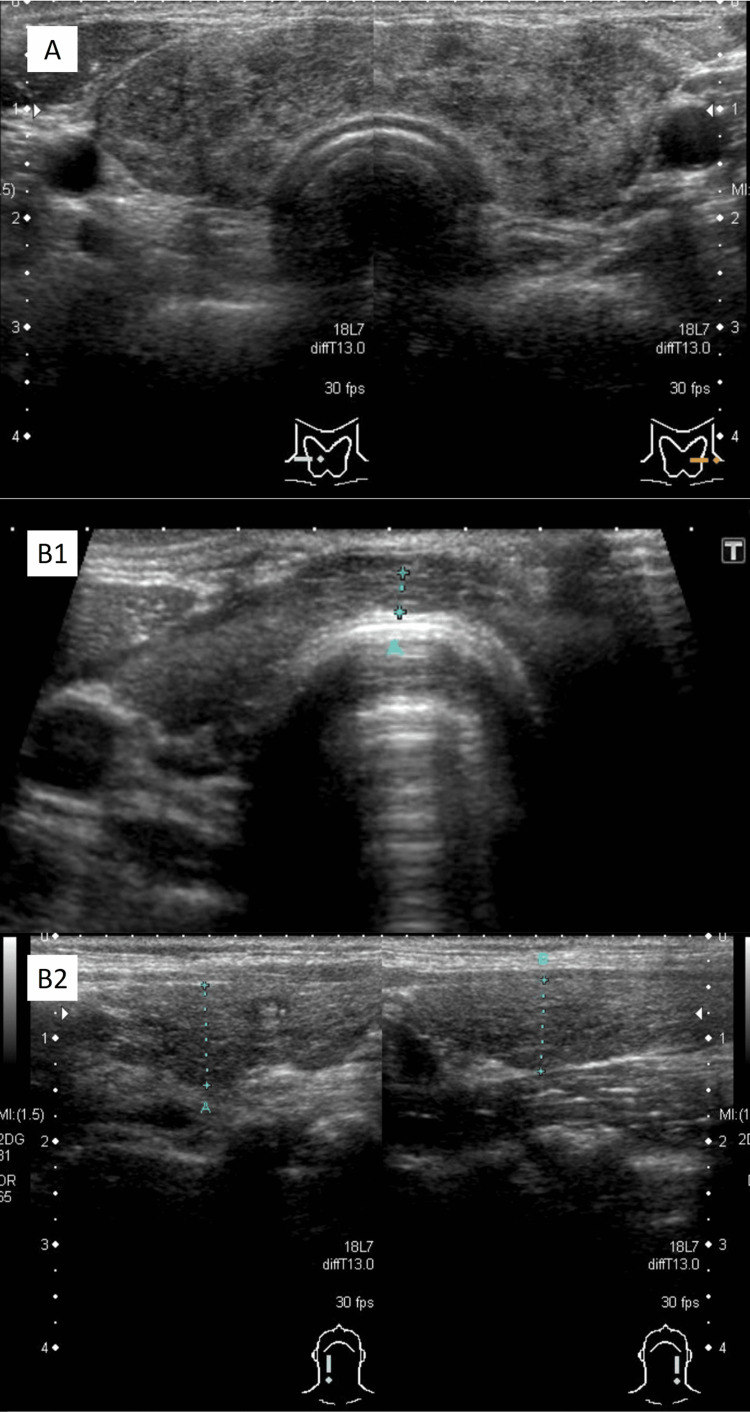
Thyroid ultrasound findings of Patient one. A: Day +103 (at onset), right lobe 19 mm, isthmus 7 mm, left lobe 19 mm B1, B2: Day +307 (at follow up), right lobe 10mm, isthmus 2 mm, left lobe 9 mm

A treatment regimen was initiated consisting of propranolol (30 mg) for heart rate control, hydrocortisone (30 mg) for supplementation to treat the relative glucocorticoid deficiency caused by thyrotoxicosis and medically induced hypoadrenocorticism, and PSL 7.5 mg for inflammation and pain. Her symptoms and thyroid enlargement improved rapidly. On day +170, the patient presented with edema and decreased cold tolerance. Thyroid hormone levels were low (FT4 <0.42 ng/dL, and TSH 89.62 μIU/mL), and accordingly, replacement therapy with levothyroxine (50 μg/day) was introduced. The dose was increased to 175 μg on day +307 and then tapered down gradually. It has been maintained at 100 μg/day since day +453. On day +307, ultrasound images showed an extremely atrophic thyroid, and anti-Tg antibody titers were elevated to 445 U/mL. Figure 1 summarizes the clinical course and Figure 2 shows the ultrasound findings.

Patient two

**Figure 3:**
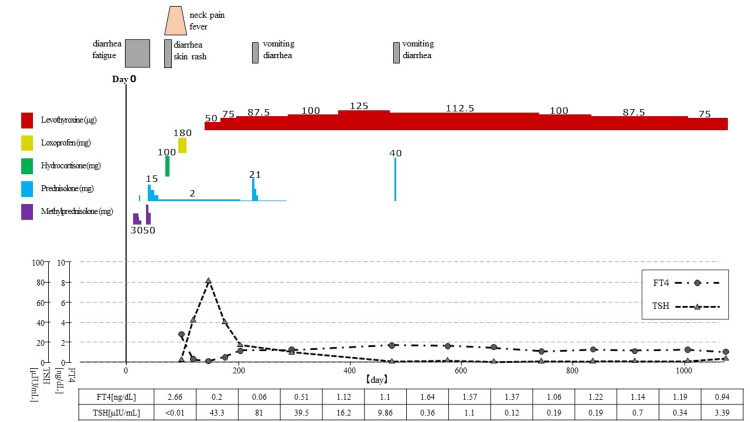
Clinical course of Patient two.

**Figure 4:**
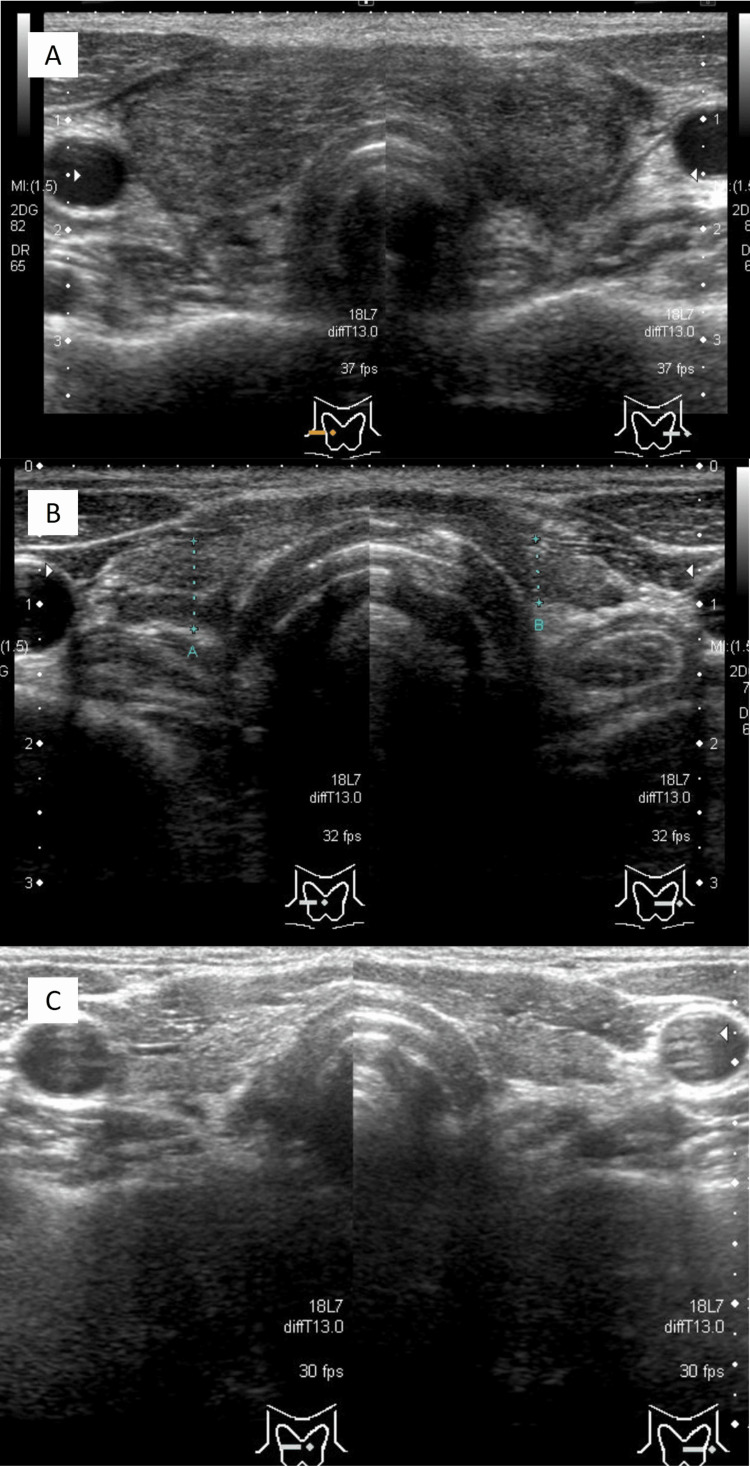
Thyroid ultrasound findings of Patient two. A:Day +99(at onset), right lobe 19 mm, isthmus 4 mm, left lobe 17 mm B:Day +293(at follow up), right lobe 6 mm, isthmus 1 mm, left lobe 5 mm C:Day +1076(at follow up), right lobe 5 mm, isthmus 1 mm, left lobe 5 mm

Laboratory tests of this 49-year-old man for fever and diarrhea showed the presence of leukopenia and blasts in peripheral blood, leading to the diagnosis of acute myelogenous leukemia. He underwent induction therapy with idamycin and cytarabine, but did not achieve remission, and was retreated with induction therapy with the T-ALL-JALSG202-O protocol. After consolidation therapy, he underwent a myeloablative conditioning regimen comprising 180 mg/m^2^ of fludarabine, 12.8 mg/m^2^ of busulfan, 80 mg/m^2^ of melphalan, and 4 Gy of TBI. CBT was performed in August 20XX. The source of cord blood was an unrelated donor with an HLA 6/6 match (C1 locus mismatch). Tacrolimus and hydrocortisone were used for GVHD prophylaxis. The patient developed diarrhea on day +17 and was diagnosed with intestinal GVHD grade one. Methylprednisolone (30 mg/day) was started and later tapered, then switched to PSL, then reduced from 5 mg to 2 mg on day +68. Fever developed around day +80. Thyroid tenderness, predominantly on the right lobe, was noted on day +93. The CRP level was elevated (11.44 mg/dL), and thyrotoxicosis was detected (FT4 2.66 ng/dL and TSH <0.01 μIU/mL). Anti-Tg antibodies were positive at 213 U/mL, whereas anti-TPO antibodies and TRAbs were negative. Thyroid ultrasonography showed a diffusely enlarged thyroid gland with a coarse inhomogeneous interior and no hypoechoic areas, masses, or increased blood flow. The patient was diagnosed with destructive thyroiditis and treated with loxoprofen for 21 days. On day +147, the patient developed hypothyroidism (FT4 0.06 ng/dL and TSH 81.0 μIU/mL), necessitating treatment with levothyroxine (50 μg/day). The levothyroxine dose was increased to 125 µg/day on day +385 and tapered gradually thereafter to maintain a steady state of euthyroidism, on 75 µg/day since day +1006. Anti-Tg antibodies were positive at 153 U/mL on day +147 but became negative at 23 U/mL by day +1076. Ultrasonography on day +293 demonstrated an extremely atrophic thyroid gland and remained atrophic on repeat ultrasonography at day +1076. Azacitidine was administered starting day +118 until day +839, as a post-treatment for acute myeloid leukemia. Figure 3 shows the clinical course of Patient two and Figure 4 shows the ultrasound images.

## Discussion

In these two cases, painful thyroiditis developed approximately 100 days after CBT. Glucocorticoids were used for acute intestinal GVHD in both cases, and painful thyroiditis developed after tapering PSL to 2-2.5 mg/day. The condition eventually progressed to hypothyroidism which required hormone replacement therapy.

Thyroiditis after allogeneic HSCT is rare. A few reports have described the development of thyroiditis after bone marrow transplantation; however, there are only very few reports of thyroiditis linked to CBT, which has become an increasingly popular treatment in recent years in Japan. To the best of our knowledge, our report is the first that describes post-CBT thyroiditis in two patients with detailed information/follow-up on thyroid function, antibody titer, and echocardiographic findings spanning >one year from disease onset.

Clinically, both cases were associated with painful goiter and thyrotoxicosis that required differentiation from subacute thyroiditis. However, hypoechoic or creeping areas, which are typically found in cases of subacute thyroiditis, were absent in both of our patients. Acute exacerbation of Hashimoto’s thyroiditis is another condition that should be ruled out in patients with painful goiter. Acute exacerbation of Hashimoto’s thyroiditis was ruled out in both of our patients since the antibody titer in Patient two was only moderately elevated, and his condition resolved rapidly following treatment with nonsteroidal anti-inflammatory drugs alone. Furthermore, the antibody titer in Patient one was also only moderately elevated, and no relapse was noted even after a fairly rapid reduction in PSL dose. Moreover, rapid and severe thyroid atrophy was noted in both patients, which is inconsistent with the typical clinical course of acute exacerbation of Hashimoto's disease.

Reports of thyrotoxicosis after allogeneic HSCT are scarce. A single-center retrospective Japanese study reported 16 cases of hypothyroidism, 28 of subclinical hypothyroidism, and only one of hyperthyroidism among 661 patients after allogeneic HSCT [3]. In a prospective study of 57 patients, Kami et al. reported eight patients who presented with transient thyrotoxicosis 61-307 days (median: 111 days) after bone marrow transplantation. Most of these cases were painless and transient, suggesting that many such cases may be undiagnosed in clinical settings [5]. With regard to the long-term course described in the above report, seven patients (excluding one who died) developed hypothyroidism within a median period of 12 months. What is the mechanism underlying the development of thyrotoxicosis after HSCT? While the exact mechanism remains elusive, one or more of the following factors may play a role; conditioning regimen, GVHD, viral infection, genetic predisposition, and autoimmunity adapted from the donor [6].

Four previous reports described eight cases of post-CBT thyrotoxicosis [6-9], all of which were Japanese patients (Table 1). The time period between CBT and thyrotoxicosis was 54 days (range, 41-217 days), which is earlier than the typical onset after bone marrow transplantation. Two of the above studies suggested that post-CBT thyrotoxicosis is due to the transfer of fetal lymphocytes contained in the cord blood and lymphocytic infiltration of the thyroid gland [6,7], similar to the phenomenon of postpartum thyroiditis. Postpartum thyroiditis causes transient thyrotoxicosis within the initial 1-3 months after delivery, followed by hypothyroidism that normalizes within one year postpartum. It is more likely to occur in women who are positive for anti-TPO antibodies, and its underlying mechanism is autoimmune thyroiditis caused by lymphocytic infiltration of the thyroid gland [10]. In our two cases, both patients were diagnosed with gastrointestinal GVHD, which was treated with glucocorticoids, and the development of thyroiditis followed a reduction in glucocorticoid dose. Both patients tested positive for anti-Tg antibodies, suggesting the involvement of immunological mechanisms. It is possible that reactivation of the immune system after immunosuppression caused by glucocorticoid reduction may have triggered the disease onset. However, in the eight previously reported cases, glucocorticoids for post-transplant GVHD were not used in two cases [6], and no details were provided in the remaining cases.

**Table 1:** Published cases of thyroiditis after cord blood transplantation. M, Male; F, Female; AML, acute myeloid leukemia; Tg, antithyroglobulin antibody; TPO, antithyroid peroxidase antibody; TSAb, thyroid-stimulating antibody; TRAb, thyroid-stimulating hormone receptor antibody

Author, Year of report	Age (yrs)	Sex	Primary disease	Onset of thyrotoxicosis	Main clinical features	Antibody	Treatment/thyroid outcome
Motohashi 2008 [8]	44	M	AML	Day 63	Pyrexia, fatigue, tremor, tachycardia	Tg(-), TPO(-), TRAb(-)	No treatment/euthyroid
Konuma 2006 [6]	32	F	AML	Day 41	Tachycardia	TPO(+)	No treatment/euthyroid
	42	M	AML	Day 42	Tachycardia	TPO(+)	No treatment/euthyroid
Konuma 2023 [9]	31	F	AML	Day 45	Pyrexia, thyroid tenderness	TPO(-), TSAb(-), TRAb(-)	β-blocker/euthyroid
	58	M	AML	Day 159	Pyrexia, thyroid tenderness	Tg(+)	None/hypothyroidism
	17	M	AML	Day116	Tachycardia	Tg(+)	β-blocker/euthyroid
	54	M	AML	Day 217	Pyrexia, thyroid tenderness	TPO(+), Tg(+)	Prednisolone (30 mg)/euthyroid
Makita 2011 [7]	12	F	AML	Day 43	Pyrexia, neck swelling, tenderness	TPO(+)	Prednisolone (20 mg)/hypothyroidism

With respect to the long-term clinical courses of our patients, levothyroxine supplementation was reduced, after reaching a peak dose at approximately 300 days in both cases. Notably, the anti-Tg antibody titer showed an increasing trend in Patient one, whereas it gradually decreased and became negative after approximately three years in Patient two. This was probably related to the use of azacitidine, which was administered as a post-treatment intervention in Patient two. It has been reported that 86% of patients with autoimmune diseases show improvement with azacitidine [11]. The mechanism of such improvement is considered to be mediated through the activation of regulatory T cells, increase in anti-inflammatory cytokines, such as interferon-gamma, suppression of CD4+ T cells, and reduction of inflammatory cytokines, such as interleukin-6 [11], suggesting that azacitidine has an immunomodulatory effect. This also implies that immunological mechanisms may be involved in post-transplant thyroiditis.

We should also discuss here the reason for the neck pain and tenderness associated with thyroiditis. We speculate that the painful thyroiditis was due to the rapid increase in thyroid gland size. This argument is based on previous reports that described the most severe pain to occur in patients who showed the largest increase in thyroid gland size [7,9].

## Conclusions

In this study, we followed the clinical courses of two adult cases of rare post-CBT painful thyroiditis, who subsequently developed thyroid atrophy and hypothyroidism. We followed both cases for more than one year and analyzed their unique clinical characteristics, response to therapy, and long-term progression. Thyroiditis with subsequent hypothyroidism should be considered in cases of anterior neck pain after CBT.
